# Psoriasis as an Immune-Mediated and Inflammatory Systemic Disease: From Pathophysiology to Novel Therapeutic Approaches

**DOI:** 10.3390/biomedicines9111511

**Published:** 2021-10-21

**Authors:** Anna Campanati, Andrea Marani, Emanuela Martina, Federico Diotallevi, Giulia Radi, Annamaria Offidani

**Affiliations:** Department of Clinical and Molecular Sciences, Polytechnic Marche University, 60100 Ancona, Italy; a.campanati@univpm.it (A.C.); andreamarani.med@yahoo.com (A.M.); federico.diotallevi@gmail.com (F.D.); radigiu1@gmail.com (G.R.); a.offidani@ospedaliriuniti.marche.it (A.O.)

**Keywords:** psoriasis, immune-mediated skin diseases, therapy, pathophysiology

## Abstract

Psoriasis is an immune-mediated inflammatory disease, with a chronic relapsing-remitting course, which affects 2–3% of the worldwide population. The progressive acquisitions of the inflammatory pathways involved in the development of psoriasis have led to the identification of the key molecules of the psoriatic inflammatory cascade. At the same time, psoriasis therapy has radically evolved with the introduction of target molecules able to modify the natural history of the disease, acting specifically on these inflammatory pathways. For these reasons, biologics have been demonstrated to be drugs able to change the disease’s natural history, as they reduce the inflammatory background to avoid irreversible organ damage and prevent systemic complications. However, several issues related to the use of biologics in patients with systemic comorbidities, remain open. All these data reflect the extraordinary potentiality of biologics, but also the unmet medical need to improve our knowledge on the long-term risk related to continuous use of these drugs, and their administration in special populations. This narrative review aims to highlight both the efficacy and safety profile of biologics in psoriasis, starting from pathophysiology and moving towards their clinical application.

## 1. Introduction

Psoriasis vulgaris is a common inflammatory, immune-mediated, chronic, and recurrent dermatosis, caused by the interplay between multiple genetic and environmental risk factors [1,2].

The clinical feature of psoriasis is dominated by erythematous-squamous plaques which usually, but non-exclusively arise symmetrically on the extensor surfaces of the elbows and knees, scalp, lumbosacral area [3], and it reflects some pathogenetic mechanisms underlying psoriasis, i.e., inflammation, hyperproliferation, angiogenesis [4]. However, current scientific evidence shows that inflammation in psoriasis involves the organism at a deeper level than skin. In moderate-to-severe psoriasis, increased levels of pro-inflammatory markers and cytokines, such as TNF-α, IL-6, IL-17, IL-23, PCR, and others, have been detected not only in skin plaques, but also in the blood and other biological fluids, such as saliva [5,6,7]. Studies performed using FDG-PET/CT proved that patients with moderate-to-severe psoriasis have subclinical inflammation in the liver, joints, and tendons, as well as global inflammation in subcutaneous tissue and arteries [8,9,10]. Furthermore, psoriasis, such as other immune-mediated inflammatory diseases i.e., rheumatoid arthritis [11], multiple sclerosis [12], and inflammatory bowel disease [13], is typically associated with comorbid conditions. Psoriatic arthritis (PsA) is the most prevalent, it develops in up to 30% of psoriatic patients [14]. Other associated conditions include, but are not limited to cardiovascular disease, diabetes mellitus, obesity, inflammatory bowel disease, and non-alcoholic fatty liver disease [15,16]. A wealth of data has recently demonstrated that psoriasis can no longer be considered a disease strictly limited to the skin, but a multisystemic disorder in which skin involvement represents the most clinically evident part of a generalized inflammatory state.

The issue is even more complex. Since the 1980s, the concept of psoriasis as a primary disorder of keratinocytes has been replaced by that of psoriasis as a disorder of the immune system, particularly of T cells [17]. Several components of the immune system are involved in the central pathogenetic mechanisms of disease, i.e., the cross-talk between innate and adaptive systems, the IL-17/IL-23 axis, the effect on resident T cells of the skin [18]. Scientific research enlightened the most crucial among them and led to the development of revolutionary targeted therapies.

Molecules like IL-17, IL-23 and TNF-α and others, act as a promoter for the immunopathogenesis development of skin lesions, but also drive the systemic involvement in course of psoriasis, and thus link-local immune-dysregulation with the generalized inflammatory state we mentioned above.

The concept of Psoriasis as a systemic immune-mediated inflammatory disease, not only changed our understanding of the origin and development of the disease but inevitably our clinical approach to the patient [19].

The purpose of this narrative overview is thus to collect data from literature, to bring out the available evidence on this “model” of psoriasis, analyzing it from various viewpoints (pathogenetic, clinical, therapeutic): a model that seems to be “avant-garde” but that is in truth realistic and, above all, necessary in clinical practice.

## 2. Materials and Methods

This narrative review was based on the general approach developed biomedical narrative review construction, that involves four key steps: 1-identify keywords; 2-conduct research; 3-review abstract and articles; 4-document results [20].

### 2.1. Identify Keywords

To identify keywords, a brainstorming approach, involving the entire research group, was used. The research team consisted of 6 dermatologists experienced in psoriasis pathophysiology. Two among them had also specific expertise in psoriasis treatment, and one in literature review methodology.

During the first meeting, the research team selected the topic, identified the scope, constructed the title, and selected the keywords as follows: “psoriasis”, “psoriasis AND immune-mediated skin diseases”, “psoriasis AND therapy” “psoriasis AND pathophysiology”.

### 2.2. Conduct Research

An extensive search for eligible articles was conducted on the following databases: National Library of Medicine PubMed database, and Scopus. The references list of selected studies was scanned to find additional records.

Inclusion criteria: studies reporting on psoriasis as immune-mediated disease, published in English language, published between 1990–2020, abstract available. No restriction on the design of the study was considered, and randomized controlled trial, case-control study, cross-sectional study, case reports and series, and review article were included.

### 2.3. Review Abstract and Article

The selection of the relevant data published in the literature took place in three steps. In the first step, three researchers (A.C., E.M. and A.M.) independently selected the articles based on the title. Any disagreement was solved by consulting a senior investigator (AO). The second step consisted of evaluating the abstracts. At least two members of the research team (F.D. and G.R.) independently assessed each abstract. The research team resolved all discrepancies through consensus.

### 2.4. Document Results

All sources with similar data/level of evidence were analyzed, collected, and grouped. The main text was structured into subsections. New evidence-based points were summarized and major points for future research and practice were defined.

## 3. Results

### 3.1. Pathophysiology of Psoriasis

A key feature of psoriasis is sustained inflammation leading to altered keratinocyte proliferation and differentiation. What triggers and maintains this inflammation is dysregulation of the immune system, both in its innate and in adaptive components, caused by the interplay between multiple genetic and environmental risk factors [21,22]. Numerous cells and molecules are involved. Among these Th1, Th17, Th9, follicular Th (Tfh) and Th22 lymphocytes and their respective cytotoxic lymphocytes, Treg, γδT cells, dendritic cells, neutrophils, mast cells, NK and NKT cells, lymphoid innate cells (ILCs), keratinocytes, and IFN-α, INF-γ, IL-17, IL-22, IL-23, TNF-α, and numerous other dendritic cell-activating molecules, autoantigens, cytokines, chemokines play a role. An exhaustive discussion about all of them is beyond the scope of this section: the most crucial for “systematic repercussion” according to current evidence, will be described.

Following a chronologic line of discussion, dendritic cells are known to play a crucial role in the early stages of the disease. Although their mechanism of activation is still unclear, data show that antimicrobial peptides (AMPs), such as LL37, -defensins, S100 proteins, and cathelicidins, secreted by keratinocytes in response to damage, activate Toll-like receptors (TLRs) expressed by plasmacytoid dendritic cells (pDC), a particular type of dendritic cells that links innate and adaptive immunity [21,23]. The activation of pDc is crucial. Triggered, they produce IFN-, which promotes the maturation of myeloid dendritic cells (mDC): a specific population of them (CD11c + CD1c- cells), under this stimulus, begins to produce molecules that have become new therapeutic targets: TNF-α, IL-23, and IL-12 [24,25,26,27,28].

TNF-α is a pleiotropic molecule, meaning it is produced by a multitude of cells in addition to the DCs, and exerts its action on a multitude of cell types. It mainly induces in these the expression of adhesion molecules and secondary mediators. Noteworthy is that it stimulates the proliferation and differentiation of T lymphocytes, Th1, Th17, and Th22, which in turn will produce TNF-α, IL-17, IL-22, favoring initiation of a self-propelled cycle of inflammation. Essentially, this cytokine plays an indirect role in disease pathogenesis by promoting adaptive immune effects of the IL-23/IL-17 axis [29,30,31,32,33].

Activation of the IL-23/IL-1 axis, the focus on which has led to revolutionary targeted therapies, determines the amplification phase of the process and the tissue cellular response. Activated dendritic cells lead to massive lymphocyte infiltration and formation of DCs/T cell clusters, that facilitate the T-mediated response. The myeloid dendritic cells that infiltrate the dermis at this stage secrete IL-23, although, like TNF-α, it is not the only cell capable of doing so [27]. IL-23 production stimulates IL-17 producing cells, which include Th17, Tc17, γδ T cells, ILC3, mast cells, and neutrophils. Noteworthy is that recent studies have revealed that most IL-17-producing cells consist of γδ T cells [27,34]. The IL-17 cytokine family consists of 6 members, A–F, but only two have a pathogenetic role in psoriasis, IL17-A, and IL-17F. The former appears to have a stronger effect than the latter [35].

IL-17, in cooperation with other cytokines such as TNF-α and IL-22, induces the development of the psoriasis phenotype through tissue cell activation. The most relevant tissue response is provided by keratinocytes that, releasing chemokines and other pro-inflammatory molecules, such as CCL20 and IL-1 F9, sustain skin inflammation. Activation of the IL-23/17 axis is thus amplified by numerous mediators, and this determines the typical gene expression profile and histopathological picture of psoriasis [27,36,37].

An inflammatory cascade begins with IFN-γ and continues with TNF-α and IL-23 to end with IL-17, with progressive “disease-specificity”, so that IL-17 inhibitors, acting further downstream, have a more rapid onset of action [38,39]. Next to these “main” mediators, we find some “collateral” ones. IL-22, for example, would be pathogenically more relevant in vitro than in vivo. The “IL-2/IFN-γ” axis, which was considered essential before the “IL-17-centric” model, deserves mention. Th1 lymphocytes activated by various mediators, including IL-12, produce IFN-γ which probably plays a role as an upstream cytokine in the IL-23/IL-17 axis, but its pharmacological inhibition has not produced satisfactory results [40]. Other cells, such as neutrophils, vascular endothelium, and macrophages, also contribute to the pathogenesis of psoriasis, through the production of molecules such as VEGF, IL-17, IL-23 [37,41,42,43]. For what concerns psoriasis as immune-mediated systemic disease some questions still are pending: What do these processes occurring in the skin microenvironment have in common, and possibly connect, with the “systemic” ones? Which are the main pathogenetic drivers in psoriasis considered as a systemic inflammatory disease? Probably the best way to find answers is starting from the inflammatory pathway analysis rather than from the canonical point of view of clinical comorbidities.

### 3.2. Relevance of IL-23/IL-17 Axis in Systemic Involvement of Psoriasis

#### 3.2.1. IL-23/IL-17 Axis and Psoriatic Arthritis

Psoriatic arthritis (PsA) is the most prevalent coexisting condition in PsO. In most cases, psoriasis precedes psoriatic arthritis [14]. Clinically, it can affect both axial and peripheral skeletons, with a wide range of clinical presentations including sacroiliitis, enthesitis, osteitis, and dactylitis.

Numerous pieces of evidence support the hypothesis that the IL-23/IL-17 axis plays a key role in the pathogenesis of PsA. High levels of Th17 cells have been found in psoriatic synovial fluid compared to rheumatic synovial fluid [44], similarly, high levels of IL-17A have been found in the synovial fluid and synovial membrane of patients with psoriatic arthritis [45]. Enthesitis, synovitis, and altered bone remodeling were observed in a mouse model after IL-23 administration. Inflammation and bone remodeling were mediated by TNF-α and IL-17 in this model [46]. Other studies have been reported in the literature on murine models with psoriasiform skin lesions, enthesitis, and arthritis were observed, and all of these models were linked to IL-23 [47].

A pathophysiologic model of psoriatic arthritis makes it resemble that of cutaneous psoriasis. A high ratio of myeloid to plasmacytoid dendritic cells was found in psoriatic synovial fluid. Inappropriate activation of dendritic cells triggers IL-23 production that activates IL-17-producing cells [48]. A second model, instead, supported by recent studies such as those mentioned above, attributes a fundamental role to the entheses, which have been proposed to be the site where the disease starts: IL-23 is released following biomechanical stress or trauma and activates IL-17 producing cells [14].

IL-17, acting in concert with other molecules produced in the process such as IL-22, IL-23, and TNF-α results in inflammation and pathological bone resorption and formation. Mesenchymal cells differentiate into osteoblasts in response to IL-22 and other signaling pathways, forming enthesophytes and syndesmophytes, involved in pathological bone formation [14]. IL-17 does not appear to directly activate osteoclasts, but stimulates osteoblasts to produce RANKL, which results, by binding to the RANK receptor on the surface of osteoclast precursors, in osteoclastogenesis [49].

We infer from current scientific evidence that both IL-17 and IL-23 play a key role in the pathogenesis of PsO and PsA, which might instead differ between skin and joints. is the “expression of the IL-23/IL-17 axis”.

For example, Belasco et al. [50] reported that gene expression patterns in skin and synovium are distinct, showing a stronger IL-17 signature in the skin than in synovium, while is an equivalent TNF-α signal across both tissues. Nerviani et al. [51] demonstrated that PsA synovial tissue shows a heterogeneous IL-23 axis profile when compared with matched skin. They reported that, while IL23A, IL12B, and IL23R are expressed at a high level in lesional skin, their expression in the synovium is hugely heterogeneous [51]. This could be the pathophysiological premise of the divergent skin-joints response, with less efficacy on the joint side, that is observed on a clinical base in patients under biological therapy. Recently, the existence of IL-23 producing cells in the axial involvement of PsA is gaining importance: the fact that IL-17 can be produced independently of IL-23, “outside the axis”, has relevant therapeutic consequences [52]. The relationship between osteoporosis, psoriasis, and psoriatic arthritis is still debated. Although these pathological conditions have common inflammatory pathways, such as TNF-α, INF-γ, IL-6, and although treatments used for PsO and PsA, such as methotrexate and cyclosporine, may induce bone rarefaction, studies on this subject are contradictory, and screening and management of osteoporosis in the psoriatic patient are still under debate [53]. A further link between osteoporosis and psoriasis could be IL-23, since this molecule induces osteoclastogenesis and receptor activator of kappa B ligand (RANKL) expression in T cells. Recent studies support the idea of a protective effect of anti-IL23 therapy, but further confirmatory studies are needed [54].

#### 3.2.2. IL-23/IL-17 Axis and Cardiovascular and Metabolic Comorbidities

The association between psoriasis and cardiovascular disease has been known for a long time, but the cause-and-effect relationship is still not well established. Robust data show that patients with severe psoriasis have an increased cardiovascular risk and reduced life expectancy. On the other hand, psoriatic patients are more likely to have a high BMI, metabolic syndrome, and type 2 diabetes mellitus. Although the contribution of cardiovascular risk factors to an increased rate of cardiovascular disease cannot be excluded, large studies show an independent relationship between PsO and cardiovascular disease [55,56,57,58].

IL-17, according to current scientific evidence, appears to be more central than IL-23 in the genesis of cardiometabolic complications in psoriatic disease.

IL-17 overexpressing mouse models had shorter life expectancy, and hypertrophic cardiomyopathy, and altered vascular endothelium compatible with increased cardiovascular risk [59]. IL-17 could have potentially opposite effects at the level of atherogenesis. IL-17 may promote the recruitment of neutrophils and monocytes to the plaque and their transformation into foam cells; at the same time, it may inhibit the expression of adhesion molecules and promote smooth muscle proliferation [60,61]. From a therapeutic point of view, instead, the CARIMA study showed that the administration of the IL-17 inhibitor secukinumab leads to a significant improvement in flow-mediated dilatation (FMD), an index of endothelial function [62]. Elnabawi et al. demonstrated that the IL-17 inhibitor was superior to biologics of other classes in reducing non-calcified plaques burden, through angiographic studies [63]. Although there are studies supporting the anti-atherogenic action of IL-17 [64,65], and the relationship between atherosclerosis and psoriasis is far from clear, overall, many authors believe it is pro-atherogenic, and, generally, the key molecule in the cardiovascular involvement of psoriasis, and as such, although still lacking controlled studies, the most promising “target” [58,66]. However, there is also a role for IL-23 in the cardiometabolic comorbidities of psoriasis, although, according to current evidence, less crucial than IL-17. The increased carotid intima-media thickness (IMT) could be considered to be a marker of generalized arteriosclerosis. Studies suggest that the carotid IMT may benefit from treatment with biological drugs, particularly anti-IL-12/23, in patients suffering from moderate-to-severe psoriasis. However, larger longitudinal studies should be performed to fully confirm these results [67].

Obesity, diabetes, and metabolic syndrome also seem to be linked to psoriasis via IL-17.

Studies show that IL-17 and cytokines secreted in response to its stimulus are relevant in the pathogenesis of obesity [68]. Obesity is associated with elevated serum levels of free fatty acids, which sensitize DCs to amplify the Th17 response [69]. In both visceral adipose tissue and other peripheral tissues of obese individuals, increased IL-17-producing T cells were detected, and elevated levels of IL-17 were found in serum [70,71].

Supporting the implication of IL-17 in the metabolic syndrome is the finding that IL-17R levels in the liver and muscle correlate with insulin resistance [72], and that blockade of IL-17 leads to decreased hepatic inflammation in non-alcoholic steatohepatitis syndrome [73].

Worthy of mention is that IL-17 interferes at the molecular level with insulin signaling. Since IL-17 activates the IκB kinase (IKK)/NFκB pathway, inhibitory phosphorylation of IRS-1 directly by IKK and indirectly by JNK activation in response to other proinflammatory cytokines is able to attenuate insulin sensitivity [74]. The opposite is also true, as shown by epidemiological studies mentioned earlier, namely that insulin resistance can worsen psoriasis. From a molecular point of view, we know that, under conditions of hyperinsulinemia, adipocytes secrete large amounts of VEGF. VEGF has been shown to activate keratinocyte inflammation, in interaction with IL-17 [75].

#### 3.2.3. IL-23/1L-17 Axis: Psoriasis between Nervous and Gastrointestinal Systems

The complex pathogenesis of psoriasis is intertwined with that of inflammatory bowel and neurodegenerative diseases, with important therapeutic implications. Psoriasis and Chron’s Disease, or Ulcerative Colitis, for example, show similarities from a pathogenesis perspective, and a higher rate of co-occurrence [76,77,78]. Again, the IL-23/IL-17 axis plays a crucial role and, again, is expressed differently than in other body sites. In “intestinal comorbidity” of psoriasis IL-23 has a decisive pathogenic role compared with IL-17.

Increased levels of IL-17 and IL-23 are found in the intestinal lamina propria of patients with Crohn’s disease. A growing body of literature demonstrates that IL-17, as opposed to IL-23, in the intestine has a role in maintaining homeostasis rather than as a driver of inflammation [79]. In murine models of intestinal disease, treatment with IL-23 inhibitors improved colic symptoms, increased the Treg/Th17 ratio, and improved epithelial barrier integrity, whereas treatment with IL-17 increased the number of proinflammatory cytokines, decreased the Treg/Th17 ratio, and worsened epithelial barrier integrity [80,81]. Manasson et al. demonstrated that in patients with psoriatic arthritis pharmacological blockade of IL-17 induced subclinical intestinal inflammation and dysbiosis [82]. Finally, gut dysbiosis could trigger IL-23-mediated inflammation, although a recent systematic review concludes that the relationship between psoriasis and the gut microbiome is still far from being understood and brought into clinical practice [14,83,84].

Court studies also report a statistically significant association between multiple sclerosis and psoriasis, an association that is denied by a few other studies [85,86,87]. The pathogenetic mechanism is unclear; however, a recent study attributing a key role to IL-17 in the genesis of neurodegenerative diseases is worthy of mention. It is able to activate microglia, astrocytes, oligodendrocyte precursors. What results is an inflammatory cascade that causes loss of dopaminergic neurons, glutamate excitotoxicity, and apoptosis of oligodendrocytes [88].

### 3.3. TNA-α in Systemic Involvement in Psoriasis: And “Old but Gold” Pathogenetic Driver

TNF-α, is the historic molecule investigated first as a pathogenic driver in psoriasis, it has demonstrated an indirect role in skin pathophysiology, promoting the effects of the IL-23/IL-17 axis.

We can state that TNF-α is a less “skin-specific” molecule, than the IL-23/IL-17 axis, but that, according to the current evidence, in psoriasis considered as a systemic inflammatory disease remains a “hallmark”.

In psoriatic arthritis, for example, its expression is stimulated by IL-17, and thus TNF-α is located “downstream” of it. TNF-α promotes pathological bone resorption by inhibiting osteoblastogenesis via Dkk-1 and promoting osteoclastogenesis via RANKL [89,90]. However, as is the case of pure cutaneous psoriasis, also the “IL-12/IFN-” axis has a pathogenic role in PsA, and TNF-α secretion, by Th1 cells, could be stimulated also in this sense. Thus, TNF-α could be located in the inflammatory cascade on the same level and upstream of IL-17 [14,89]. Although its position may seem collateral, “around” the IL-23/IL-17 axis, in reality, its role is crucial. TNF-α is equally expressed in the synovial tissue of psoriatic arthritis and rheumatoid arthritis [44]. According to Belasco et al. IL-17 is expressed proportionally more in psoriatic skin than in the joint, whereas TNF-α expression is equivalent in the two [50]. A question arises: can we consider IL-17 a “more cutaneous” molecule and TNF-α a “more arthropathic” molecule, in the context of psoriasis considered as a systemic disease, as suggested by the brilliant efficacy of TNF-α inhibitors in joint symptomatology and their superiority over IL-17/23 inhibitor, highlighted by studies?

The same is true for cardiometabolic involvement. In the process of atherosclerotic plaque formation, TNF-α plays a fundamental role. Armstrong et al. [90], noted how there might be two links between inflammation in psoriasis and atherogenesis. The first, mentioned above, is driven by the IL-23/17 axis, with a prominent role of IL-17, which is, according to the authors, involved in plaque instability. The second is, again, driven by the IL-12/INF-γ axis, with activation of Th1 cells producing TNF-α, which is more involved in plaque development. A study has demonstrated, through an interesting bioinformatics approach, that the dominant pro-inflammatory signals linking atherosclerosis and psoriasis are that of TNF-α and INF-γ [91]. Globally, apart from psoriatic disease, TNF-α is known to induce insulin resistance both in vitro and in vivo, by reducing tyrosine kinase activity of the insulin receptor, and endothelial dysfunction, and it may contribute to altered cardiac remodeling after myocardial infarction. TNF-α exacerbates hepatic insulin resistance, resulting in increased FFA synthesis and decreased FFA oxidation, thereby promoting hepatic steatosis. It contributes to the pro-inflammatory state of obesity [11,92].

Numerous pieces of evidence estimate that in inflammatory bowel diseases (IBD), local TNF-α secretion induces not only tissue damage but also activation of the adaptive immune system, which perpetuates the inflammatory state resulting in systemic inflammation [93]. Although clinical trials with nonselective anti-TNF-α antibodies completely failed, the role of TNF-α in the pathogenesis of multiple sclerosis is still widely debated [94].

### 3.4. Other Molecules, Other Cells: A Currently Collateral Role in Systemic Involvement of PsO

Studies have highlighted the role of other molecules and cells in the pathogenesis of psoriasis as an immune-mediated systemic inflammatory disease. According to the current evidence, however, they appear collateral or dependent on the systems considered above.

IL-22 has a role in the joint and cardiometabolic involvement of psoriasis, but is essentially dependent on the IL-23/IL-17 axis, since it has a primarily cooperative action with IL-17 [14,90]. Moreover, the strategy of blocking IL-22 has proven to be not effective in treating psoriasis [27]. L-1β induces dermal γδ T cell proliferation and IL-17 production in mice. In addition, IL-1β stimulates keratinocytes to secrete chemokines that preferentially chemoattract peripheral CD27- CCR6 + IL-17 capable of producing γδ T cells (γδT17) [95].

Adipokines are cytokines produced by adipose tissue, which have functions in the regulation of metabolic functions, such as glucose and lipid metabolism, inflammation, and vascular homeostasis. They have been implicated in cardiovascular involvement in psoriasis. According to a recent review on the subject by Lynch et al. [96], studies on adiponectin are contradictory regarding its pattern in PsO, and prospective controlled studies are needed to clarify their relationship. High levels of pro-inflammatory cytokines resistin and leptin have been detected and correlate with disease severity, but rather than a pathogenic role they may constitute markers of disease.

Vitamin D is a regulator of keratinocyte differentiation, and low levels of vitamin D have been associated with metabolic syndrome and increased cardiovascular risk. However, studies in this regard suggest a need to treat low serum levels of vitamin D in the course of psoriasis, rather than a central pathogenic role of vitamin D in psoriasis as a systemic disease [97].

Communication between neutrophils and macrophages is crucial in any inflammatory response and especially that underlying atherosclerosis [98], and NETosis, or the ability of neutrophils to expel cytosolic and nuclear material forming extracellular traps that ensnare extracellular microbes, has been proposed to have a role in atherosclerosis as well as psoriasis, but these findings seem too general [98,99].

Further studies are certainly needed to further investigate the role in the systemic involvement of psoriasis of other cells and molecules than IL-17, IL-23, and TNF-α, which currently seem “more central”.

### 3.5. Other Links and Further Complexity in “Systemic PsO”

Scientific research has revealed other and increasingly complex associations of cutaneous psoriasis with other extracutaneous conditions. Psoriatic patients have a higher incidence and prevalence of uveitis, and the involvement of IL-17, IL-23, TNF-α, and IL-6 molecules unite both conditions [100,101]. Asthma and psoriasis can coexist and what they have in common is IL-17A [102]. Psoriasis is also linked to polycystic ovary syndrome and it has been shown that skin clinical features can vary depending on the PCOS phenotype [103]. A recent meta-analysis found that patients with psoriasis appear to have a slightly increased risk of cancer, particularly keratinocyte cancer and lymphomas, although data for National and International Registries on treatment with biologic agents did not show an increased risk of cancer, and data on cancer in patients with psoriatic arthritis remain inconclusive [104]. The prevalence of having depression or anxiety is higher in psoriasis patients than in controls, and TNF-α could be a pathophysiologic link between the two conditions [105,106].

## 4. Discussion

### 4.1. Need for an Integrated Multidisciplinary Care of the Psoriatic Patient

Research and models focused on the pathogenesis of psoriasis as a systemic immune-mediated inflammatory disease should not remain speculative and self-referential, rather they should lead to a more coherent and comprehensive model of the clinical approach to the psoriatic patient. Research groups, such as the Group for Research and Assessment of Psoriasis and Psoriatic Arthritis (GRAPPA), now emphasize the concept of “psoriatic disease” rather than psoriasis [107,108]. An integrated approach to the psoriatic patient is needed today, which has been shown by various studies to improve patient care [107,108,109,110,111].

The management of the psoriatic patient today raises strictly practical problems in daily clinical practice. Pre-therapeutic check-ups before starting biological or systemic therapies, which usually consist of instrumental and routine laboratory tests, sometimes lead to detection of abnormalities requiring specialized extra-dermatological evaluation. Moreover, the multidisciplinary approach to psoriasis usually leads to finding the most appropriate treatment for any specific patient.

For example, in psoriatic arthritis, nonsteroidal anti-inflammatory drugs (NSAIDs) are effective in improving joint symptoms, especially in case of the mild disease. However, in case of even mild skin involvement, early treatment with conventional synthetic disease-modifying anti-rheumatic drugs (csDMARDs) should be considered [107,108].

The multidisciplinary approach represents the best way to answer the most frequent questions regarding which are the most appropriate blood and instrumental tests to frame the patient as a whole, and which is the therapy most appropriate considering all the systemic features of psoriasis.

For these reasons, several integrated derma-gastro-rheumatological out-patients assessment modes have been set up in clinical settings.

According to this model, the patient is examined simultaneously by several specialists and submitted to specific questionnaires, e.g., on quality of life or joint symptoms, additional examinations such as ultrasound [US] with power Doppler [PD], plain X-ray, and magnetic resonance imaging [MRI], requested if necessary, and therapy discussed and shared by all of them before being prescribed. In our experience this represents a feasible and sustainable approach, and that it leads to an improvement in the management of psoriasis, an amelioration of quality of life for patients, and not at least, an improvement of clinical skills for physicians, based on an increase in mutual specific knowledge [112].

### 4.2. Patient Tailored Treatment

Psoriasis therapy consists of a “step-wise approach” of both topical and systemic treatments: topical therapy is mainly indicated in mild psoriasis, systemic therapy in moderate-to-severe form. Topical therapy and phototherapy, although they can provide effective relief of localized skin symptoms, they do little to affect underlying disease causes [113]. Considering psoriasis as a systemic immune-mediated inflammatory disease, the use of systemic therapy, able to “go beyond” the skin to reach other organs involved, is greatly increased comparing 20 years ago.

The most important clinical implication deriving from the identification of the inflammatory pathways that underlie psoriasis and its comorbidities is that the paradigm of their treatment has changed. Rather than simply treat each “comorbidity” with a specific drug, the idea to inhibit a specific inflammatory pathway to treat psoriasis and simultaneously prevent any extra-cutaneous comorbidities has made its way over the past 10 years [114].

Table 1 shows preferred biological drugs according to specific systemic comorbidities.

#### 4.2.1. Inhibiting TNF−α: From Psoriatic Arthritis to Coexisting Multiple Sclerosis

We previously highlighted the importance of TNF-α in the systemic involvement of psoriasis at every level: therefore, it is not surprising that its inhibitors are indicated, in cases of patients with other inflammatory “comorbidities” (i.e., IBD, PsA, uveitis) Moreover, TNF-α inhibitors entered the market before others, and therefore the amount of data supporting their efficacy and safety profile is wider.

Regarding articular involvement, TNF-α inhibitors act by suppressing bone resorption mediated by TNF-α. They demonstrated significant improvement in both joint symptomatology and radiologically assessed structural damage [116,117]. Infliximab, adalimumab, golimumab, and certolizumab pegol have shown comparable efficacy and are now all approved for the treatment of PsA [118,119,120]. In addition, current data demonstrated that TNF-α inhibitors are more effective than IL-12/IL-23 inhibitor ustekinumab [121]. Bonifati and Graceffa reported 7 patients who experienced worsening or a flare of psoriatic arthritis after they were switched from TNF-α inhibitors to ustekinumab, suggesting that ustekinumab has better performance on skin symptoms than on joint inflammation [122].

According to experts, TNF-α inhibitors are preferred agents if cardiovascular risk factors can be detected. TNF-α is certainly a crucial molecule in the process of atherosclerosis. However, the relationship between these drugs and cardiometabolic syndrome is complex and not yet fully understood.

Wu and Poon found that psoriatic patients treated with TNF-α inhibitors were at lower risk of myocardial infarction than untreated patients [123]. Psoriatic patients who do not respond to biologics show a minimal reduction in MACE (Major Adverse Cardiovascular Events) risk [124,125,126]. A large retrospective US study with information from over 7.5 million patients with a mean follow-up time of 4.7 years found individuals with psoriasis who received TNF-α inhibitors had a lower risk for major cardiovascular events than those receiving oral/phototherapy or topical therapy [123]. Another study has shown that adalimumab therapy leads to a reduction in markers of endothelial activation and intima-media thickness, but few preliminary findings suggest clinical significance [127,128]. Nonetheless, studies on the relationship between TNF-α inhibitors and congestive heart failure are contradictory. The New York Heart Association recommendations are as follows: TNF-α inhibitors are contraindicated in class 3 or 4 CHF; echocardiogram should be done before treatment initiation in class 1 or 2 CHF and TNF-α inhibitors should be avoided in patients with ejection fraction 50%; TNF-α inhibitors should be discontinued in patients with new-onset CHF [126,129].

TNF-α inhibitors are not contraindicated in type 2 diabetes mellitus and/or insulin resistance. However, there are no conclusive data to enunciate meaningful clinical recommendations. Although some studies suggest that TNF-α inhibitors are associated with a decrease in insulin resistance, the study by Wu et al. has shown no benefit with anti-TNF-α drugs in combination with methotrexate versus methotrexate alone on HbA1C or fasting blood glucose in psoriasis patients [130,131,132,133]. There is no evidence to show whether one of the listed drugs is efficacious compared with another in a patient with psoriasis and fatty liver disease [134].

TNF-α inhibitors are not contraindicated in obese patients. Infliximab should be preferred as its dose is based on weight. However, since a few studies have detected the possibility of weight gain during treatment with TNF-α inhibitors, IL-17, IL12-23, and IL23 inhibitors appear currently more indicated [135].

The use of anti-TNF-α in case of coexistence with other immune-mediated and inflammatory diseases such as IBD, depression, multiple sclerosis, is also complex. They are considered the first-line treatment in the case of moderate-severe Chron’s Disease, apart from etanercept that has not shown the same efficacy, perhaps due to different pharmacodynamics [136]. Conversely, TNF-α inhibitors are absolutely contraindicated in the case of multiple sclerosis [137]. These drugs can be used with caution if there is a coexistence of Lupus, while caution should be applied in case of coexisting malignant tumor pathology [115]. Strober et al. evaluated the efficacy of infliximab, etanercept, and adalimumab on depression in psoriasis patients utilizing Psoriasis Longitudinal Assessment and Registry (PSOLAR). The study showed that, compared with conventional therapy, biologics reduced the risk for depressive symptoms whereas phototherapy did not [138].

#### 4.2.2. Inhibiting the “IL-23/IL-17 Axis”: Targeting IL-17 or IL-23

We previously discussed the importance of the IL-23/IL-17 axis in the systemic involvement of psoriasis, although the two key molecules, IL-17 and IL-23, might play different roles and thus constitute different therapeutic targets.

Regarding joint involvement, we mentioned that both IL-17 and IL-23, according to current evidence, appear to have a crucial pathogenetic role. Phase 3 studies showed that IL-17 inhibitors secukinumab and ixekizumab are effective in both symptom control and radiological disease progression [139,140]. Furthermore, it is expert opinion that IL-23 inhibitors guselkumab, tildrakizumab, and risankizumab are effective in PsA patients, although they are unlicensed in this indication [141]. However, the issue is more complex. Recently, in fact, IL-23-independent IL-17-producing cells have drawn attention to the pathogenesis of axial involvement. PsA and ankylosing spondylitis (AS) are recognized as one of spondyloarthritis (SpA) characterized by enthesitis, peripheral and axial arthritis, and negative laboratory results of rheumatoid factor and anti-cyclic citrullinated peptide antibody. It indicates that the responsiveness of a certain drug for AS reflects its responsiveness for axial involvement of PsA. Anti-IL-17 antibodies showed efficacy for AS, while anti-IL-23 antibodies did not. This suggests that axial involvement in psoriatic arthritis would be better treated with IL-17 inhibitors instead of IL-23 inhibitors, although further data is needed [142].

The molecules appear to perform different functions at the level of the gut. IL-17 seems to be implicated in the maintenance of homeostasis and epithelial barrier integrity; IL-23 is then the pathogenic driver. According to current scientific evidence, whereas IL-17 inhibition exacerbates inflammatory bowel disease, IL-23 inhibitors demonstrated clinical improvement and so they indicated in the case of co-existence of Chron Disease [143,144].

Conversely, IL-17 has so far shown a greater pathogenic role than IL-23 in cardiometabolic involvement. A study revealed that hyperglycemia is highly associated with psoriasis, mainly through IL-17. In patients, the severity of psoriasis correlated with high blood glucose levels, and anti-IL-17A monoclonal antibody therapy reduced HbA1c levels significantly in these patients [143]. IL-17 inhibitors are highly effective regardless of a patient’s weight but are shown to have even better clearance rates in non-obese patients. Although there is not enough data on IL-23 inhibitors according to the patient’s weight, preliminary data seem to demonstrate they are highly efficacious agents, nonetheless. The IL-23/17 axis inhibitors thus appear more indicated than anti-TNF-α antibodies in obesity [115].

Promising data demonstrating that anti-IL-17 antibodies may have a cardiovascular risk-reducing effect in psoriatic patients have been recently published. Elnabawi et al. conducted a prospective, observational study in order to investigate the effect of biologic therapy on coronary artery plaque [63]. After 1 year of biologic therapy, patients treated with IL-17 inhibitors showed a 12% reduction in non-calcified coronary plaque burden, which was greater than that of patients treated with ustekinumab and those not treated with biologics. In the ustekinumab study (VIP-U), 12 weeks of treatment reduced aortic vascular inflammation by 6.6% versus a 12.1% increase with placebo (*p* = 0.001). However, no differences in aortic vascular inflammation were observed after 52 weeks of ustekinumab treatment [145]. Additional data is certainly needed to explore the cardioprotective effect of IL-17 and IL-23 antibodies.

Finally, we can state that IL-17 inhibitors can be administered in patients with multiple sclerosis, while data are lacking for IL-23 inhibitors, although there are no case reports of MS worsening [146]. Both classes of drugs can be administered in case of congestive heart failure [115]. Data are limited for IL-17 and IL-23 inhibitors in casa of co-existence of malignancy, and more long-term studies are needed to assess their carcinogenic potential [115,134].

### 4.3. Biomarkers Availability for Therapeutic Effect Monitoring

As psoriasis can be considered a systemic immune-mediated inflammatory disease, it is fair to ask about the existence of biomarkers to monitor this systemic involvement which can be used in clinical practice as diagnostic, prognostic, and therapeutic efficacy tools.

To be defined as a biomarker, a molecule needs to pass validation tests. For instance, a good biomarker must have a high prognostic and predictive value.

Many molecules have been advocated as biomarkers of psoriasis, first and foremost serum molecules. Among them is C-reactive protein (CRP), whose elevation can be considered an independent risk factor for cardiovascular risk (as levels of CRP decrease, cardiovascular risk lowers) [147] and whose elevation is associated both with psoriasis and psoriatic arthritis [148]. Studies demonstrated that biological therapy decreases blood CRP levels in both psoriasis and coexisting conditions. In patients with moderate-to-severe psoriasis treated with systemic therapies, including adalimumab, etanercept, infliximab, and ixekizumab, studies have reported reductions in erythrocyte sedimentation rate (ESR) and/or CRP levels [149,150,151,152]. TNF-α inhibitors also significantly reduce CRP levels in patients who have either metabolic syndrome or Crohn’s disease [153]. IL-17A inhibitor secukinumab reduces ESR levels in PsA [154]. Etanercept therapy was associated with significant reductions in transaminases, CRP, and fasting insulin, and an increase in insulin sensitivity, whereas treatment with PUVA did not lead to significant reductions in these markers [155].

A long list could be made of candidate serum molecules to be biomarkers. those of greatest clinical interest are IFN-γ, IL-6, IL-8, IL-12 [5], whose serum levels are systematically increased in serum of psoriasis patients. Despite its pathogenetic importance, studies of serum IL-17 elevation are contradictory [6,156]. Chandran et al. identified a combination of serum markers (increased levels of high sensitivity CRP, osteoprotegerin, matrix metalloproteinase 3, and the CPII:C2C ratio) that could distinguish PsA and PsO patients, demonstrating the efficacy of integrating multiple markers [157]. Circulating levels of Th1, Th17, and Th22 cells are increased in psoriasis patients compared to healthy volunteers, and the frequency of Th1 and Th17 cells is decreased after anti-TNF-α therapy [158]. Other molecules studied were antimicrobial proteins such as S100A7 (psoriasin), S100A8 (calgranulin A), and S100A9 (calgranulin B), neuropeptides, adipokines [159]. Possible genetic, transcriptional, and tissue markers from translational research have also been investigated. For instance, polymorphisms in TNAFAIP3, a gene encoding for a zinc finger protein (A20), are associated with response to anti-TNF-α agents [160]. CXCL10 is a possible biomarker for the development of psoriatic arthritis among patients with psoriasis [161].

None of the above-reported molecules achieved regulatory approval and has entered clinical routine application. The strictly inherent answer to the crucial question: “are there biomarkers for psoriasis? is then “no”, as no validate biomarkers are currently available. Instead, there is strong evidence that a treatment targeting “key cytokines” has an effect “beyond the skin”, i.e., on generalized inflammation and systemic involvement, and so the answer could be “yes”, if questioning the possibility of changing the history of the disease and its systemic involvement, especially in the case of “early” intervention [162].

## 5. Need for an Integrated Model of Psoriasis: Conclusions and Future Perspectives

There is still a long way to go in research on this subject. Starting from this awareness, in this review we simply tried to change perspective. That is, we attempted to abandon, for a moment, the concept of psoriasis as a skin disease associated with comorbidities. We have researched and assembled the current scientific evidence into a discourse that, from beginning to end, i.e., from pathophysiology to therapy, place the skin on the same level as other organs, or, better yet, unify the skin with other organs into a single body, the whole body, i.e., the presumed real target of PsO.

Instead of describing the organ-specific pathophysiology, we tried to deeply evaluate the molecular axes leading to immune-mediated inflammation and how this develops in the various organ. This approach implies that instead of identifying the most appropriate drug for a given comorbidity, it seems reasonable to try to understand what it means to inhibit these molecular axes in the various organs, also according to a multidisciplinary model of care. The progression from psoriasis to its comorbidities has been called “psoriatic march”. In this model psoriasis and atherosclerosis share common underlying immunologic mechanisms, in which Th1 and Th17 cells release TNF-α, IFN-γ, IL-17, IL-22, which contribute to both hyperproliferation and activation of keratinocytes and to the growth and instability of psoriatic plaque. Obesity is associated with low-grade systemic inflammation because it drives metabolic and immunological pathways including Th17 cell differentiation. Moreover, obesity generates biomechanical stress at joints and entheses and triggers, via IL-23, psoriatic arthritis worsening [163].

From the current scientific evidence emerges a picture characterized by the presence of main molecular axes, namely IL-23/IL-17 and TNF-α, which can be considered the pathogenic drivers of disease both at a cutaneous and systemic level, and they become revolutionary therapy targets. However, the matter is more complex, since there are many other molecules that “revolve around” these axes, such as IL-22, moreover both IL-23/IL-17 and TNF-α axes are expressed differently among the various organs affected. IL-17 seems to be more implicated than IL-23 in cardiovascular involvement, whereas in intestinal involvement the opposite is true. IL-17 and IL-23 are crucial molecules in both psoriatic skin and joint but, for instance, Nerviani et al. showed that IL-23 is expressed differently between the skin and affected joints, with higher expression of IL-23A, IL-23R, and IL-12B in the skin than in the synovium, providing a possible explanation to divergent outcomes in PsA clinical trials whereby IL-23 have better results in the skin than in joints [51]. This has relevant pathogenic and therapeutic implications, since it affects the choice of biological therapy that may be prescribed. We still do not have a precise psoriatic biomarker with diagnostic and prognostic value, but we have numerous evidence, including inflammatory molecules quantifiable in blood, that give us confirmation that biological therapy can change the history of psoriasis as a systemic immune-mediated inflammatory disease, that is, for example, it can reduce cardiovascular damage. Finally, the need for a multidisciplinary approach to the disease emerges.

So, if we scientifically look very far from where we are now, we can see in the “omic” era, a large amount of data produced by different new technologies will be decrypted, analyzed, and extracted. Databases are going to be deconvoluted by translational bioinformatics, and data integrated with other data from other sources. Thus, data of different types, i.e., gene expression, gene variant, proteomics, epigenetics, are going to be integrated with clinical, diagnostic, and therapeutic data in the next future. Psoriasis may no longer be “a skin disease associated with comorbidity”, but psoriatic patients will be grouped into “molecular subtypes” of the systemic disease named psoriasis. A subtype could differ from the others for its molecular pathophysiology, i.e., for a different type of activation of inflammatory cascades or for its type of gene or epigenetic expression. Every patient could differ from the others from a clinical point of view, but not because it simply has a different type of skin lesion, but because it will have a certain type and a certain degree of cutaneous, articular, cardiovascular, and otherwise systemic involvement.

Furthermore, patients could be classified according to the expression of specific and precise diagnostic biomarkers and therapeutic response predictors.

All these considerations could have significant therapeutic repercussions moving clinicians toward the “patient-tailored” treatment, according to the “precision medicine model”, with systemic action. In the next future patient suffering from psoriasis will be followed by a dermatologist with this cultural and scientific background.

All this seems to be at least very far, however, meanwhile, closer to us concrete questions seeking an answer are arising, stimulating research towards them.

Can there be a decisive role of other molecules, besides IL-17, IL-23, and TNF-α, in the pathogenesis of psoriasis as IMIDs? And if not, how might these axes be differently expressed, in the context of a psoriasis case history? What is the relationship, or rather what is the boundary, between psoriasis and other diseases that may coexist? What are the precise links between the skin and other rarer comorbidities, such as uveitis? Can there be reliable biomarkers of psoriasis? Can we be able to accurately quantify the contribution of psoriasis in cardiovascular risk, beyond cumulative risk? What other differences, in terms of efficacy, clinical indication, and safety, are there between biologic drugs, beyond those already known? And probably others not mentioned.

We are still far from understanding the entire “Psoriatic Universe”, there is a long way to go. As the exploration of the pathogenesis of psoriasis progresses, the complex relationship between psoriasis and its comorbidities becomes more understandable, and probably the best way to proceed towards the most complete understanding of the disease is to let the pathogenetic mechanisms guide us towards the clinical management of the patient.

## Figures and Tables

**Table 1:** Preferred biologic drugs according to specific systemic comorbidities (modified from Kaushik et al. [115]).

Drug	PsA	CD	Cancer	Obesity	CR	CHF	MS	SLE
Etanercept	++	+	−	+	++	−/+	X	+/−
Adalimumab	++	++	−	+	++	−/+	X	+/−
Infliximab	++	++	−	++	++	−/+	X	+/−
Certolizumab Pegol	++	++	−	+	++	−/+	X	+/−
Ustekinumab	+	++	+	++	+	++	+	+
Secukinumab	++	−	?/+	++	?	++	+	?/+
Ixekizumab	++	−	?/+	++	?	++	+	?/+
Brodalumab	+	−	?/+	++	?	++	+	?/+
Guselkumab	?	+	?/+	++	?	++	?/+	?/+
Tildrakizumab	?	+	?/+	++	?	++	?/+	?/+
Risankizumab	?	+	?/+	?	?	++	?/+	?/+

PsA: psoriatic arthritis; CD = Crohn’s disease; CR = Cardiovascular risk; CHF: Congestive Heart Failure; MS = Multiple Sclerosis, SLE = Systemic Lupus Erythematosus. Two plus symbols (++) indicates preferred agents; one plus symbol (+) indicates that the agent can be used; one plus symbol and one minus symbol (+/−) indicate that the drug can be used but is controversial; one minus symbol and one plus symbol (−/+) indicates that the drug is not preferred but can be used; one question mark and one plus symbol (?/+) indicates that there are not enough data but that the drug is likely safe to use; one question mark (?) indicates that there are not enough data; one minus symbol (−) indicates that use of that drug is controversial because there are not enough data; and X indicates that a drug is contraindicated.

## Data Availability

Not applicable.
